# A Case of SMARCB1-Deficient Sinonasal Carcinoma With Clear Cell Morphology

**DOI:** 10.7759/cureus.59684

**Published:** 2024-05-05

**Authors:** Tomoko Tamaki, Kyonosuke Teruya, Hitoshi Hirakawa, Mariko Tomita, Naoki Wada

**Affiliations:** 1 Department of Diagnostic Pathology, University of the Ryukyus Hospital, Nishihara, JPN; 2 Department of Otorhinolaryngology, Head and Neck Surgery, Graduate School of Medicine, University of the Ryukyus, Nishihara, JPN; 3 Department of Pathology and Oncology, Graduate School of Medicine, University of the Ryukyus, Nishihara, JPN

**Keywords:** clear cell, carcinoma, sinonasal, ini-1, smarcb1

## Abstract

SMARCB1 is a gene known to cause carcinogenesis in many soft tissue tumors, including malignant rhabdoid tumors and epithelioid sarcoma. Since the first report of a subtype of sinonasal carcinoma characterized by a deficiency of the SMARCB1 gene in 2014 to date, fewer than 200 cases have been reported. We report a case of SMARCB1-deficient sinonasal carcinoma with clear cell morphology. In our case, there are no evident basaloid or plasmacytoid/rhabdoid tumor cells, which are typical histopathological features of SMARCB1-deficient sinonasal carcinoma. SMARCB1-deficient sinonasal carcinoma is prone to recurrence and has a very poor prognosis. As the development of molecularly targeted agents progresses, therapeutic efficacy is expected to improve. Simultaneously, the importance of early and accurate diagnosis of SMARCB1-deficient sinonasal carcinoma will increase. With the limited information provided by biopsy specimens, it is necessary to confirm the loss of SMARCB1 expression by immunohistochemistry and investigate the presence of SMARCB1 gene deletion by molecular genetics, considering the possibility of SMARCB1-deficient sinonasal carcinoma even in atypical cases without basaloid or plasmacytoid/rhabdoid cell morphology, as in our case.

## Introduction

Sinonasal carcinoma is a relatively rare cancer, accounting for approximately 3-5% of all head and neck carcinomas. The incidence is less than one case per 100,000 people per year worldwide [1,2]. Among these, independent German and American researchers first described a subtype of sinonasal carcinoma characterized by a deficiency of the SMARCB1 gene in 2014 [3,4]. It is now called SMARCB1-deficient sinonasal carcinoma or switch/sucrose nonfermenting (SWI/SNF) complex-deficient sinonasal carcinoma [5]. SMARCB1 is a gene known to cause carcinogenesis in many soft tissue tumors, including malignant rhabdoid tumors and epithelioid sarcoma. SMARCB1-deficient sinonasal carcinoma is extremely rare, and according to Bell et al. [6], only 3.3% (16 cases) of the 484 reported paranasal sinus tumors were SMARCB1-deficient sinonasal carcinoma. Currently, less than 200 cases of SMARCB1-deficient sinonasal carcinoma have been reported. Not only is SMARCB1-deficient sinonasal carcinoma a rare disease but also most lesions are diagnosed at the locally advanced pT4 stage, are prone to recurrence, and have a very poor prognosis [7]. However, optimal treatment has not yet been established. More case studies are needed to understand this tumor better and determine the optimal treatment. We encountered a case of SMARCB1-deficient sinonasal carcinoma with clear cell morphology. In our case, there were no evident basaloid or plasmacytoid/rhabdoid tumor cells, which were typical histopathological features of SMARCB1-deficient sinonasal carcinoma. Therefore, we report this case, including a literature review.

## Case presentation

**Figure 1:**
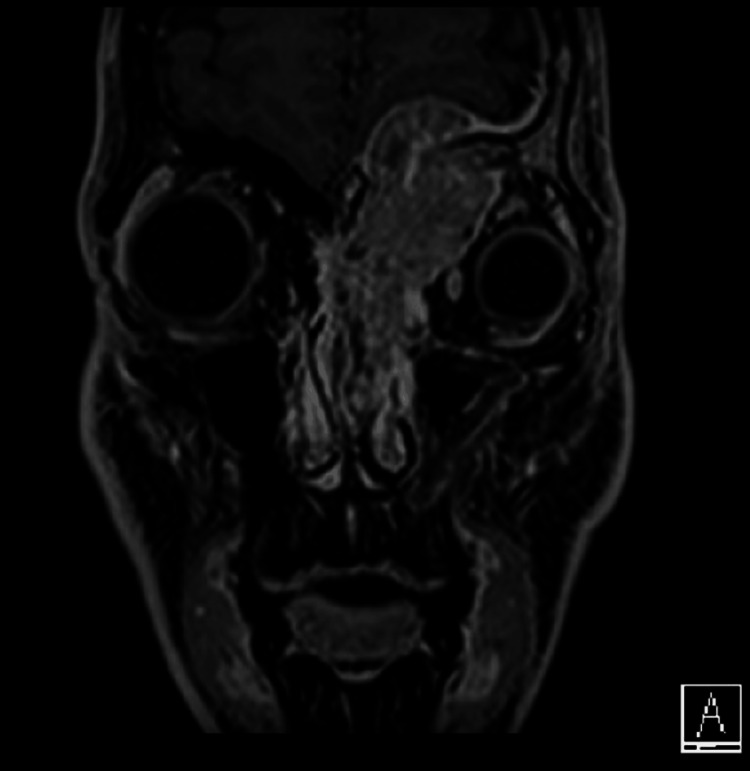
Magnetic resonance imaging (MRI) on admission. The tumor was mainly located in the left ethmoid sinus and filled the nasal cavity. It destroyed the basal skull bone and invaded the dura mater on the cranial side, compressing the brain parenchyma. Additionally, it significantly extended into the left orbit, causing eyeball displacement.

The patient was a 29-year-old male. He complained of pain around the left eye and diplopia on the upward gaze. A mass lesion involving the left nasal cavity and ethmoid sinus was observed (Figure 1).

Cranially, the lesion extended beyond the anterior skull base into the dura mater. Externally, the lesion extended into the orbit. The tumor grew relatively rapidly and was inoperable because of further extension from the left superior orbital fissure into the cavernous sinus. Radical chemoradiotherapy (70 gray (Gy) radiation + cisplatin × 3 courses) was administered, and a complete response was achieved. However, three months after completion of chemoradiotherapy, he complained of neck pain, and a local recurrence of the primary tumor was observed. Bone metastasis on the left side of the C3 cervical vertebra was also observed. RANMARK subcutaneous injection (Daiichi Sankyo Company, LIMITED, Tokyo, Japan) was started for bone metastasis, and palliative irradiation was started for the C3 cervical vertebral metastasis. After irradiation, the patient received systemic chemotherapy (CBDCA + 5-FU + pembrolizumab × 3 courses), but the disease progressed. The chemotherapy was changed to paclitaxel and cetuximab, but multiple lung metastases were observed, and the patient died 12 months after the onset of symptoms.

**Figure 2:**
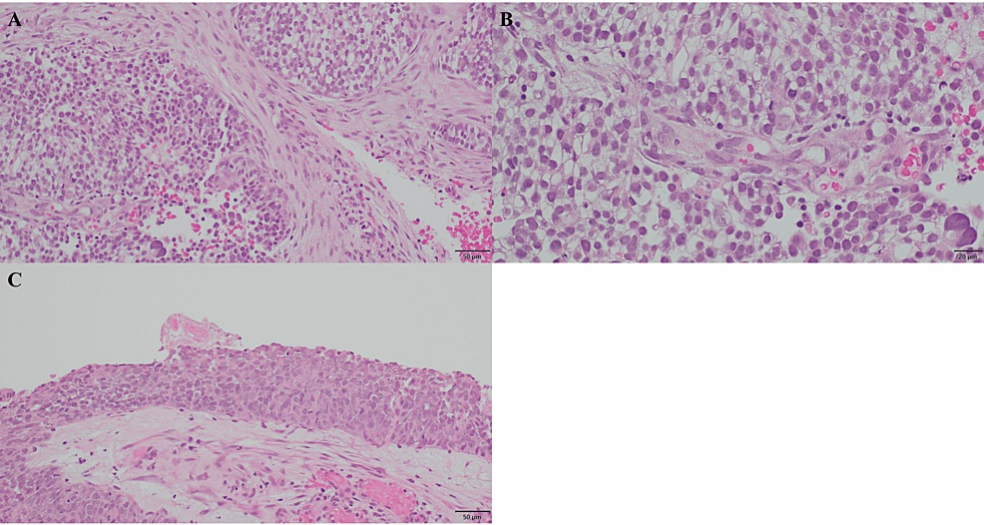
Hematoxylin and eosin staining of pathologic examination. (A) The tumor cells infiltrated and increased in a sheet-like and nest-like pattern (objective ×20). (B) The invasive tumor cells were atypical cells with clear cytoplasm, enlarged nucleus, and well-defined nucleolus. There were no evident basaloid, rhabdoid, or plasmacytoid invasive tumor cells (objective ×40). (C) In the background of invasive carcinoma, intraepithelial carcinoma was found (objective ×20).

Hematoxylin and eosin staining revealed atypical cells with clear cytoplasm, enlarged nucleus, and well-defined nucleolus infiltrating and increasing in a sheet-like and nest-like pattern (Figures 2A-2B).

**Figure 3:**
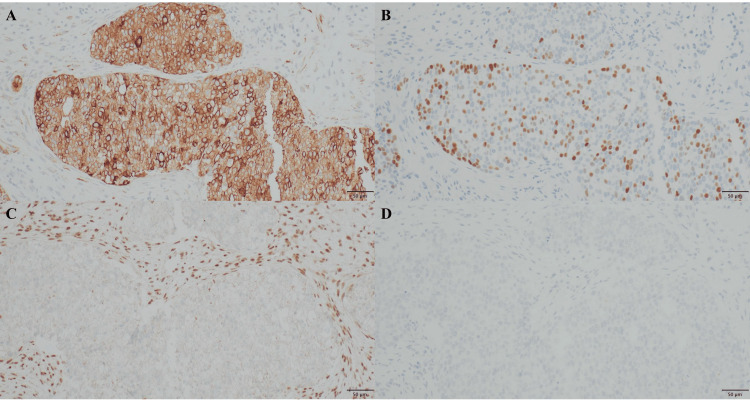
Immunostaining of pathologic examination. The tumor cells were diffuse positive for cytokeratin AE1/AE3 (A), partial positive for p40 (B), and negative for INI-1 (C) and CA-IX (D) (objective ×20).

The invasive growth of basaloid tumor cells was indistinct, and there were no evident rhabdoid or plasmacytoid tumor cells. In the background of invasive carcinoma, intraepithelial carcinoma, in which tumor cells proliferated throughout the coated epithelial layer, was found (Figure 2C). Immunostaining showed diffuse positivity for cytokeratin AE1/AE3, partial or faint positivity for p40, p63, synaptophysin, and chromogranin A, and negativity for INI-1, CA-IX, PAX-8, CD56, and NUT (Figures 3A-3D).

Based on these findings, a diagnosis of SMARCB1-deficient sinonasal carcinoma was made.

Comprehensive genomic profiling was performed using FoundationOne® CDx (F1CDx) (Foundation Medicine Inc., Cambridge, MA) to detect cancer-associated gene mutations, including the SMARCB1 gene deletion. The F1CDx technology is a next-generation sequencing (NGS)-based cancer genome profiling test previously reported by Frampton et al. [8]. Briefly, after obtaining formalin-fixed paraffin-embedded samples from surgical specimens, 20 unstained sections of 4 µm thickness and one HE-stained section were submitted to Foundation Medicine Inc. F1CDx used NGS to analyze the entire coding DNA of 324 cancer-related genes and also assessed both tumor mutation burden (TMB) and microsatellite instability (MSI). F1CDx detected the deletion of exons from 5 to 9 of the SMARCB1 gene, consistent with the immunostaining result. Additionally, deletions of the androgen receptor gene and single nucleotide substitutions in the CREBBP (T1939S), CTCF (N349S), PRDM1 (R683Q), and RAD21 (P278L) genes were found. However, all of these mutations were of unknown pathologic significance. Additionally, TMB and MSI could not be determined.

## Discussion

Histopathologically, SMARCB1-deficient sinonasal carcinoma is characterized by sheet-like, solid, or alveolar growth of tumor cells with basaloid or plasmacytoid/rhabdoid cell morphology. According to a report by Lee et al. [7], regarding the cell morphology of SMARCB1-deficient sinonasal carcinoma, basaloid cell morphology was the most common cell morphology (68/192 cases, 35.4%), followed by plasmacytoid/rhabdoid cell morphology (37/192 cases, 19.3%). These two cell morphologies accounted for approximately half of all cases. The clear cell morphology observed in our case was present locally alongside basaloid cell morphology in one case (0.5%) and was also present locally alongside plasmacytoid/rhabdoid cell morphology in another case (0.5%).

In our case, no evident basaloid or plasmacytoid/rhabdoid cell morphology was observed, and the tumor cells had clear cell morphology, making it difficult to suspect a SMARCB1-deficient sinonasal carcinoma. The loss of SMARCB1 protein expression was confirmed by using immunostaining technique. We also attempted to analyze the deletion of the SMARCB1 gene, but we could not get significant results because of insufficient sample volume to perform the multiplex ligation-dependent probe amplification (MLPA) assay (data not shown). However, comprehensive genomic profiling using F1CDx revealed the deletion in exons from 5 to 9 of the SMARCB1 gene, providing genetic support for the immunostaining result. Several genetic mutations were detected, but all these mutations had unknown pathological significance, and no genetic mutations associated with carcinogenesis were found other than the deletion of the SMARCB1 gene. The immunostaining results, including INI-1 in our case, were also consistent with SMARCB1-deficient sinonasal carcinoma [5]. Although clear cell morphology was present, immunostaining was negative for CA-IX and PAX-8, confirming the negative results for metastatic clear cell renal cell carcinoma. Since the patient was male, there was also no possibility of metastatic ovarian clear cell carcinoma, one of the representative carcinomas with clear cell morphology. Additionally, according to the WHO Classification of Tumours, Female Genital Tumours (5th ed.), about 40-50% of ovarian clear cell carcinoma cases harbor loss-of-function mutations in ARID1A, a tumor suppressor and part of the SWI/SNF chromatin remodeling complex. It might lead to clear cell morphology to destabilize the activity of the SWI/SNF complex in some carcinomas. More detailed analyses are needed to elucidate the mechanism of leading clear cell morphology, and it might be a good strategy to focus on the destabilization of the SWI/SNF complex activity.

In most of the previous reports, SMARCB1 expression defects were confirmed by immunostaining alone (104 of 128 cases). As far as we know, to date, 24 cases have been reported and analyzed using additional molecular genetic techniques. These included nine cases of fluorescence in situ hybridization (FISH) [4,9], two cases of NGS [7,10], one case of FISH and NGS [11], four cases of NGS and in-situ hybridization [12], and eight cases of NGS and MLPA [13]. Two of these cases showed no abnormalities on FISH [4]. These two cases were histological of low-grade malignancy and were initially diagnosed as myoepithelial carcinoma [4]. Previously, SMARCB1-deficient myoepithelial carcinoma of soft tissue was reported to have an EWSR1 gene rearrangement located on the same chromosome 22 as SMARCB1 without SMARCB1 gene abnormalities [14]. These two cases, wherein FISH detected no EWSR1 and SMARCB1 gene abnormalities, might represent a different tumor type from SMARCB1-deficient sinonasal carcinoma. Although SMARCB1 expression disappeared by immunohistochemistry, the two cases without SMARCB1 gene aberrations by FISH might suggest that another inactivating event below the resolution of FISH occurred. Thus, the small number of cases without apparent SMARCB1 gene deletion on FISH might involve a different oncogenic mechanism from a typical case with apparent SMARCB1 gene deletion on FISH, which might affect histologic grade and prognosis. The outcome at the time of reporting these two cases was that one patient had died of the present disease after 17 months of observation, while the other was alive after 26 months of observation. On the other hand, in SMARCB1-deficient sinonasal carcinoma, most lesions are diagnosed at the locally advanced pT4 stage, are prone to recurrence, and have a very poor prognosis [7]. Therefore, it is advisable to perform a molecular genetic search in addition to an immunohistochemical search to determine whether the lesion is a typical SMARCB1-deficient sinonasal carcinoma wherein both searches are abnormal.

The SMARCB1 gene is a known tumor suppressor gene located on chromosome 22q11.2. The gene product, SMARCB1 or INI-1, is expressed in the nucleus of normal cells as a core subunit of the SWI/SNF chromatin remodeling complex. The SWI/SNF complex acts as an antagonist of enhancer of zeste homolog 2 (EZH2). EZH2 alters the structure of chromatin and represses tumor suppressor genes by adding a methyl group to the 27th lysine of histone H3. A deficiency of SMARCB1 leads to the destabilization of the activity of the SWI/SNF complex, which loses its ability to antagonize EZH2. Thereby, enhancement of EZH2 activity reduces the expression of tumor suppressor genes, which ultimately upregulates oncogenic pathways [15]. To date, no standard treatment for SMARCB1-deficient sinonasal carcinoma has been established. Combinations of different therapies have been applied, such as surgery, chemotherapy, and radiotherapy. However, SMARCB1-deficient sinonasal carcinoma is highly invasive and has a very poor prognosis. Therefore, the development of molecular targets and novel therapies are needed.

EZH2 inhibitors are promising therapeutic candidates for SMARCB1-deficient sinonasal carcinoma. It has been reported that suppressing EZH2 activity restores the expression of tumor suppressor genes and prevents cancer cell growth by inducing cell cycle arrest and apoptosis. Tazemetostat, a drug designed to inhibit the EZH2 activity, has the potential to be effective against many tumors including SMARCB1-deficient tumors [16-19]. It has become an approved drug in the United States for treating SMARCB1-deficient epithelioid sarcoma [20]. Currently, it is also subjected to clinical trials overseas to treat locally advanced SMARCB1-deficient sinonasal carcinoma.

As the development of molecularly targeted agents progresses, therapeutic efficacy is expected to improve. Simultaneously, the importance of early and accurate diagnosis of SMARCB1-deficient sinonasal carcinoma will increase. With the limited information provided by biopsy specimens, it is necessary to confirm the loss of SMARCB1 expression by immunohistochemistry and to investigate the presence of SMARCB1 gene deletion by molecular genetics, considering the possibility of SMARCB1-deficient sinonasal carcinoma even in atypical cases without basaloid or plasmacytoid/rhabdoid cell morphology, as is our case.

## Conclusions

Herein, we report an extremely rare case of SMARCB1-deficient sinonasal carcinoma with clear cell morphology. In our case, there are no evident basaloid or plasmacytoid/rhabdoid tumor cells, which are typical histopathologic features of SMARCB1-deficient sinonasal carcinoma. SMARCB1-deficient sinonasal carcinoma is a newly recognized subtype of sinonasal carcinoma with unique clinicopathologic features that make accurate diagnosis difficult, especially in small biopsy specimens. More case data and studies are needed to define the clinical and pathologic features and to improve treatment modalities. To this end, combining morphologic findings with molecular genetic testing is essential to characterize the molecular details further and accurately identify the disease. Because SMARCB1-deficient sinonasal carcinoma is progressive and has a poor prognosis, it is necessary to develop targeted therapeutics against SMARCB1-associated molecular abnormalities. This paper provides valuable information for accurately diagnosing this rare cancer and understanding its molecular mechanisms.

## References

[1] Turner JH, Reh DD (2012). Incidence and survival in patients with sinonasal cancer: a historical analysis of population-based data. Head Neck.

[2] Fuoco J, Huang M, Esfandiari N (2024). SMARCB1 (INI1)-deficient sinonasal carcinoma manifesting as oral lesions: a report of two cases. Head Neck.

[3] Agaimy A, Koch M, Lell M (2014). SMARCB1(INI1)-deficient sinonasal basaloid carcinoma: a novel member of the expanding family of SMARCB1-deficient neoplasms. Am J Surg Pathol.

[4] Bishop JA, Antonescu CR, Westra WH (2014). SMARCB1 (INI-1)-deficient carcinomas of the sinonasal tract. Am J Surg Pathol.

[5] Agaimy A, Din N, Jain D, Rooper L, Bal M, Mittal N (2023). SWI/SNF complex-deficient sinonasal carcinoma. WHO Classification of Tumours Editorial Board. Head and neck tumours.

[6] Bell D, Hanna EY, Agaimy A, Weissferdt A (2015). Reappraisal of sinonasal undifferentiated carcinoma: SMARCB1 (INI1)-deficient sinonasal carcinoma: a single-institution experience. Virchows Arch.

[7] Lee VH, Tsang RK, Lo AW (2022). SMARCB1 (INI-1)-deficient sinonasal carcinoma: a systematic review and pooled analysis of treatment outcomes. Cancers (Basel).

[8] Frampton GM, Fichtenholtz A, Otto GA (2013). Development and validation of a clinical cancer genomic profiling test based on massively parallel DNA sequencing. Nat Biotechnol.

[9] Su YJ, Lee YH, Hsieh MS (2022). SMARCB1(INI1)-deficient sinonasal adenocarcinoma: report of a case previously diagnosed as high-grade non-intestinal-type sinonasal adenocarcinoma. Pathol Int.

[10] Gomez-Acevedo H, Patterson JD, Sardar S, Gokden M, Das BC, Ussery DW, Rodriguez A (2019). SMARC-B1 deficient sinonasal carcinoma metastasis to the brain with next generation sequencing data: a case report of perineural invasion progressing to leptomeningeal invasion. BMC Cancer.

[11] Jamshidi F, Pleasance E, Li Y (2014). Diagnostic value of next-generation sequencing in an unusual sphenoid tumor. Oncologist.

[12] Dogan S, Chute DJ, Xu B (2017). Frequent IDH2 R172 mutations in undifferentiated and poorly-differentiated sinonasal carcinomas. J Pathol.

[13] Libera L, Ottini G, Sahnane N (2021). Methylation drivers and prognostic implications in sinonasal poorly differentiated carcinomas. Cancers (Basel).

[14] Le Loarer F, Zhang L, Fletcher CD (2014). Consistent SMARCB1 homozygous deletions in epithelioid sarcoma and in a subset of myoepithelial carcinomas can be reliably detected by FISH in archival material. Genes Chromosomes Cancer.

[15] Yamaguchi H, Hung MC (2014). Regulation and role of EZH2 in cancer. Cancer Res Treat.

[16] Italiano A, Soria JC, Toulmonde M (2018). Tazemetostat, an EZH2 inhibitor, in relapsed or refractory B-cell non-Hodgkin lymphoma and advanced solid tumours: a first-in-human, open-label, phase 1 study. Lancet Oncol.

[17] Morschhauser F, Tilly H, Chaidos A (2020). Tazemetostat for patients with relapsed or refractory follicular lymphoma: an open-label, single-arm, multicentre, phase 2 trial. Lancet Oncol.

[18] Gounder M, Schöffski P, Jones RL (2020). Tazemetostat in advanced epithelioid sarcoma with loss of INI1/SMARCB1: an international, open-label, phase 2 basket study. Lancet Oncol.

[19] Vejmelkova K, Pokorna P, Noskova K (2023). Tazemetostat in the therapy of pediatric INI1-negative malignant rhabdoid tumors. Sci Rep.

[20] Cooper GW, Hong AL (2022). SMARCB1-deficient cancers: novel molecular insights and therapeutic vulnerabilities. Cancers (Basel).

